# Polyunsaturated fatty acids and risk of Alzheimer’s disease: a Mendelian randomization study

**DOI:** 10.1007/s00394-019-02126-x

**Published:** 2019-11-01

**Authors:** Yasutake Tomata, Susanna C. Larsson, Sara Hägg

**Affiliations:** 1 Department of Medical Epidemiology and Biostatistics, Karolinska Institute, Nobels väg 12A, 17 156 Stockholm, Sweden; 2 Division of Epidemiology, Department of Health Informatics and Public Health, Tohoku University School of Public Health, Graduate School of Medicine, Sendai, Japan; 3 Department of Surgical Sciences, Uppsala University, Uppsala, Sweden

**Keywords:** Polyunsaturated fatty acids, Alzheimer’s disease, Mendelian randomization analysis

## Abstract

**Purpose:**

Observational studies have suggested that polyunsaturated fatty acids (PUFAs) may decrease Alzheimer’s disease (AD) risk. In the present study, we examined this hypothesis using a Mendelian randomization analysis.

**Methods:**

We used summary statistics data for single-nucleotide polymorphisms associated with plasma levels of n-6 PUFAs (linoleic acid, arachidonic acid) and n-3 PUFAs (alpha-linolenic acid, eicosapentaenoic acid, docosapentaenoic acid, docosahexaenoic acid), and the corresponding data for AD from a genome-wide association meta-analysis of 63,926 individuals (21,982 diagnosed AD cases, 41,944 controls).

**Results:**

None of the genetically predicted PUFAs was significantly associated with AD risk; odds ratios (95% confidence interval) per 1 SD increase in PUFA levels were 0.98 (0.93, 1.03) for linoleic acid, 1.01 (0.98, 1.05) for arachidonic acid, 0.96 (0.88, 1.06) for alpha-linolenic acid, 1.03 (0.93, 1.13) for eicosapentaenoic acid, 1.03 (0.97, 1.09) for docosapentaenoic acid, and 1.01 (0.81, 1.25) for docosahexaenoic acid.

**Conclusions:**

This study did not support the hypothesis that PUFAs decrease AD risk.

**Electronic supplementary material:**

The online version of this article (10.1007/s00394-019-02126-x) contains supplementary material, which is available to authorized users.

## Introduction

The overall results from observational studies suggest that daily intake of polyunsaturated fatty acids (PUFAs) is inversely associated with risk of incident Alzheimer’s disease (AD) and cognitive impairment [1]. Previous prospective studies of PUFA biomarkers have also supported the preventable association [2, 3].

However, observational studies are generally susceptible to methodological problems such as confounding. Therefore, randomized controlled trials (RCTs) are more suitable to examine causal relationships. However, no RCT has yet reported the preventive effect of PUFAs on incident AD among cognitively normal adults [4].

To overcome the problem of confounding in observational studies, Mendelian randomization (MR) is becoming widespread for assessing causal relationships. MR study is a type of instrumental variable analysis where genetic variants (single-nucleotide polymorphisms: SNPs) are applied as instrumental variables for the potential risk factor. MR study is often described as a “natural RCT” because random allocation of alleles during meiosis is conceptually similar to the RCT design. Therefore, a MR study would provide more robust evidence regarding the causal relationship between PUFAs and AD than observational studies. However, to our knowledge, no MR study has yet investigated this relationship.

The aim of the present MR study was to examine the hypothesis that higher plasma levels of PUFAs decrease AD risk. We examined the main six types of PUFAs, including the n-6 PUFAs linoleic acid (LA) and arachidonic acid (AA) and the n-3 PUFAs alpha-linolenic acid (ALA), eicosapentaenoic acid (EPA), docosapentaenoic acid (DPA), and docosahexaenoic acid (DHA).

## Methods

### Study design

To estimate causal associations between lifelong exposures to PUFA levels and the risk of late-onset AD, we conducted a two-sample MR analysis using summary statistics data from genome-wide association studies (GWAS).

### Summary statistics data for PUFAs

We used summary statistics data from the Cohorts for Heart and Aging Research in Genomic Epidemiology (CHARGE) consortium examining the association between genetic variants and plasma levels of n-6 PUFAs [5] and n-3 PUFAs [6]. These summary statistics were based on a meta-analysis of GWAS of individuals of European ancestry (*n* = 8631 individuals for n-6 PUFAs and 8866 individuals for n-3 PUFAs). Age ranged from 21 to 102 years [6].

### Summary statistics data for AD

We used two types of summary statistics data for AD.

First, to conduct primary analysis, we used data from GWAS for the association between genetic variants and clinically diagnosed late-onset AD (Table S1) [7]. The summary statistics data was based on a meta-analysis of GWAS from four consortia comprising a total of 63,926 individuals of European ancestry (21,982 AD cases and 41,944 cognitively normal controls). The mean age at AD onset ranged from 71.1 to 82.6 years [7].

Second, to conduct secondary analysis, we used summary statistics data from another GWAS for the association between genetic variants and AD including AD-by-proxy based on parental diagnoses (Table S2) [8]. The summary statistics data was based on a meta-analysis consisting of a total of 455,258 individuals of European ancestry (71,880 AD cases, 383,378 controls). The AD-by-proxy phenotype showed a strong genetic correlation with AD (*r*_g_ = 0.81) [8].

### Selection of instrumental variables

We selected 8 SNPs associated with one or more of the PUFAs (Table S1). All SNPs satisfied the following conditions: (1) associated with the PUFAs at genome-wide significance (*P* < 5 × 10^−8^); (2) biologically relevant to PUFAs (involved in PUFA metabolism); (3) strongest association within the specific locus (e.g., among *FADS1*) [5, 6]. In addition, the same SNPs have been used as instrumental variables for PUFAs in previous MR studies, and were not considered as weak instrumental variables (*F* statistic ≥ 10) [9].

### Mendelian randomization analysis

For each PUFA, two-sample MR analyses were performed to calculate the odds ratios and 95% confidence intervals for AD. Odds ratios were shown as per one standard deviation increase based on standard deviations of the largest study (the Atherosclerosis Risk in Communities Study) in the CHARGE consortium (Table S3) [6, 10]. All MR analyses were performed by using the “MendelianRandomization” package in R 3.5 (R Project for Statistical Computing). We mainly used the command for the inverse variance weighted method “mr_ivw”. In addition, we also conducted sensitivity analyses using the weighted median method and the MR-Egger regression method for PUFAs for which at least 3 SNPs were available as instrumental variables (i.e., LA and DPA). The weighted median approach provides a consistent estimate for a causal effect if at least 50% of the information in the analysis comes from variants that are valid instrumental variables. The MR-Egger regression method estimates the effect size by adjusting for horizontal pleiotropy. We tested for pleiotropy using the MR-Egger regression intercept. This test is based on the assumption that the intercept (the gene–outcome association) should be zero if the gene–exposure association is zero.

### Ethical issues

Because the present study is based on GWAS summary statistics rather than individual level data, an ethical approval is not required according to the rules at Karolinska Institutet, Sweden.

## Results

**Fig. 1:**
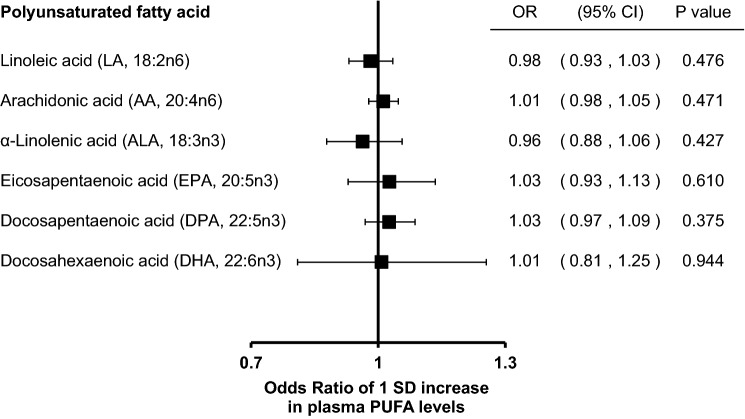
Mendelian randomization results: phospholipid levels of polyunsaturated fatty acids and Alzheimer’s disease (21,982 clinically diagnosed cases and 41,944 controls)

The results of the MR analyses based on the inverse variance weighted method are shown in Fig. 1. All genetically predicted plasma PUFA levels were not statistically significantly associated with AD risk.

Results obtained by the weighted median and MR-Egger regression analyses (Table S4) were not essentially different from those based on the inverse variance weighted method (Fig. 1). Plots to visualize the results of the analyses based on the MR-Egger method are shown in Figure S1 and Figure S2. There was no evidence for pleiotropy based on the MR-Egger regression analyses (*P*-intercept > 0.05).

Results from the secondary analyses (Table S5) were consistent with the main findings (Fig. 1).

## Discussion

To our knowledge, this is the first MR study to examine the relationship between genetically predicted levels of PUFAs and AD. Our results showed that none of the PUFAs was statistically significantly associated with AD risk. Results from our MR study, which is less prone to confounding compared with observational studies, did not support previous results from observational studies showing that PUFAs are inversely associated with AD risk.

As major methodological issues about bias towards the null in MR studies, weak instrument bias and low statistical power are often suggested. Although we only used 1–3 SNPs as instrumental variables for each PUFA, the SNPs explained a relatively large variation in PUFA levels and they fulfilled the criterion as not being weak instrumental variables (*F* statistic > 10) [9]. Additionally, to increase statistical power, we conducted secondary analyses based on a larger dataset including AD-by-proxy based on parental diagnoses (Table S5). Results of these analyses (Table S5) were consistent with the results of the main analysis (Fig. 1). Therefore, weak instrument bias and low statistical power are unlikely to explain our main results.

Regardless of statistical power, effect sizes (point estimates) were smaller in the current MR study than in previous observational studies. For example, a recent cohort study reported that the hazard ratio per 1 SD increment of red blood cell EPA + DHA levels for dementia was 0.92 (*P* < 0.05) [3]. Furthermore, results of a meta-analysis of cohort studies showed that a 0.1-g/day increment of dietary DHA intake was significantly associated with a lower risk of AD (relative risk = 0.63, *P* < 0.001) [1]. However, a subgroup analysis suggested that other dietary factors (e.g., vitamin E intake) were regarded as residual confounding factors [1]. Therefore, the difference in results between the present MR study and previous observational studies might be explained by residual confounding rather than methodological issues in the MR design such as weak instrument bias.

The present study has several limitations. First, we only used 1–3 SNPs as instrumental variables for each PUFA. Therefore, regression estimates such as pleiotropy test by MR-Egger regression might not be robust. Second, because shared SNPs were used among the PUFAs (e.g., rs174547 was used for LA, AA, ALA, and DPA), identifying the effects for individual PUFAs was difficult.

In conclusion, the present MR study did not support the hypothesis that PUFAs decrease AD risk.

## Electronic supplementary material

Below is the link to the electronic supplementary material.

Supplementary material 1 (DOCX 370 kb)

## Abbreviations

PUFA: Polyunsaturated fatty acid
AD: Alzheimer’s disease
RCT: Randomized controlled trial
MR: Mendelian randomization
LA: Linoleic acid
AA: Arachidonic acid
ALA: Alpha-linolenic acid
EPA: Eicosapentaenoic acid
DPA: Docosapentaenoic acid
DHA: Docosahexaenoic acid
GWAS: Genome-wide association study
