# Supplementary material for: Polyunsaturated fatty acids and risk of Alzheimer’s disease: a Mendelian randomization study
Source: Eur J Nutr. 2019 Nov 1;59(4):1763–6. doi: 10.1007/s00394-019-02126-x (PMC7230050; doi:10.1007/s00394-019-02126-x)
Supplement: Supplementary file 1 — Supplementary material 1 (DOCX 370 kb) [file 394_2019_2126_MOESM1_ESM.docx]

**Table S1.** Summary statistics of phospholipid levels of polyunsaturated fatty acids-raising genetic variants

|  | | | | | **Effect size estimates for PUFAs^a^** | | | | | |  | **Effect size estimates for AD^b^** | | |
| --- | --- | --- | --- | --- | --- | --- | --- | --- | --- | --- | --- | --- | --- | --- |
| **PUFA** | **SNP** | **Chr** | **Effect allele** | **Other allele** | **EAF** | ***β^c^*** | **SE** | **p** | **%VE per allele** | **%VE per IV** |  | ***β*** | **SE** | **p** |
| Linoleic acid (LA, 18:2n6) | rs10740118 | 10 | G | C | 0.56 | 0.248 | 0.043 | 8.08*10^-9^ | 0.2–0.7 | 8.3–21.3 |  | 0.0158 | 0.0144 | 0.272 |
|  | rs174547 | 11 | C | T | 0.32 | 1.474 | 0.042 | 4.98*10^-274^ | 7.6–18.1 |  |  | -0.0120 | 0.0151 | 0.424 |
|  | rs16966952 | 16 | G | A | 0.69 | 0.351 | 0.044 | 1.23*10^-15^ | 0.5–2.5 |  |  | -0.0094 | 0.0155 | 0.544 |
| Arachidonic acid (AA, 20:4n6) | rs174547 | 11 | T | C | 0.68 | 1.691 | 0.025 | 3.00*10^-971^ | 32.63 | 33.07 |  | 0.0120 | 0.0151 | 0.424 |
|  | rs16966952 | 16 | G | A | 0.69 | 0.199 | 0.031 | 2.43*10^-10^ | 0.44 |  |  | -0.0094 | 0.0155 | 0.544 |
| α-Linolenic acid (ALA, 18:3n3) | rs174547 | 11 | C | T | 0.33 | 0.016 | 0.001 | 3.47*10^-64^ | 1.03 | 1.03 |  | -0.0120 | 0.0151 | 0.424 |
| Eicosapentaenoic acid (EPA, 20:5n3) | rs3798713 | 6 | C | G | 0.43 | 0.035 | 0.005 | 1.93*10^-12^ | 0.36 | 2.05 |  | 0.0016 | 0.0144 | 0.910 |
|  | rs174538 | 11 | G | A | 0.72 | 0.083 | 0.005 | 5.37*10^-58^ | 1.69 |  |  | 0.0079 | 0.0155 | 0.608 |
| Docosapentaenoic acid (DPA, 22:5n3) | rs780094 | 2 | T | C | 0.41 | 0.017 | 0.003 | 9.04*10^-9^ | 0.46 | 11.58 |  | 0.0177 | 0.0145 | 0.222 |
|  | rs3734398 | 6 | C | T | 0.43 | 0.040 | 0.003 | 9.61*10^-44^ | 2.74 |  |  | -0.0011 | 0.0143 | 0.939 |
|  | rs174547 | 11 | T | C | 0.67 | 0.075 | 0.003 | 3.79*10^-154^ | 8.38 |  |  | 0.0120 | 0.0151 | 0.424 |
| Docosahexaenoic acid (DHA, 22:6n3) | rs2236212 | 6 | G | C | 0.57 | 0.113 | 0.014 | 1.26*10^-15^ | 0.65 | 0.65 |  | 0.0010 | 0.0143 | 0.945 |
| key: Chr, chromosome; EAF, effect allele frequency; IV, instrumental variable; PUFA, polyunsaturated fatty acid; SE, standard error; SNP, single-nucleotide polymorphism; VE, Variation explained | | | | | | | | | | | | | | |
| ^a^ Summary statistics for PUFA from “PLoS Genet 2011;7(7):e1002193. doi: 10.1371/journal.pgen.1002193” and “Circ Cardiovasc Genet 2014;7(3):321-31. doi: 10.1161/CIRCGENETICS.113.000208” | | | | | | | | | | | | | | |
| ^b^ Summary statistics for clinically diagnosed late-onset AD (21,982 cases, 41,944 controls) from “Nat Genet 2019;51(3):414-30. doi: 10.1038/s41588-019-0358-2”.  ^c^ Expressed as % of total fatty acids. | | | | | | | | | | | | | | |

**Table S2.** Summary statistics of AD for secondary analysis (including AD-by-proxy based parental diagnoses: 71,880 cases and 383,378 controls)

|  | | | |  | **Effect size estimates for AD^a^** | | |
| --- | --- | --- | --- | --- | --- | --- | --- |
| **PUFA** | **SNP** | **Effect allele** | **Other allele** |  | ***β*** | **SE** | **p** |
| Linoleic acid (LA, 18:2n6) | rs10740118 | G | C |  | 0.00331 | 0.00213 | 0.120 |
|  | rs174547 | C | T |  | -0.00161 | 0.00223 | 0.469 |
|  | rs16966952 | G | A |  | 0.00042 | 0.00231 | 0.857 |
| Arachidonic acid (AA, 20:4n6) | rs174547 | T | C |  | 0.00161 | 0.00223 | 0.469 |
|  | rs16966952 | G | A |  | 0.00042 | 0.00231 | 0.857 |
| α-Linolenic acid (ALA, 18:3n3) | rs174547 | C | T |  | -0.00161 | 0.00223 | 0.469 |
| Eicosapentaenoic acid (EPA, 20:5n3) | rs3798713 | C | G |  | -0.00003 | 0.00212 | 0.990 |
|  | rs174538 | G | A |  | 0.00058 | 0.00230 | 0.801 |
| Docosapentaenoic acid (DPA, 22:5n3) | rs780094 | T | C |  | -0.00005 | 0.00215 | 0.982 |
|  | rs3734398 | C | T |  | 0.00044 | 0.00212 | 0.835 |
|  | rs174547 | T | C |  | 0.00161 | 0.00223 | 0.469 |
| Docosahexaenoic acid (DHA, 22:6n3) | rs2236212 | G | C |  | -0.00031 | 0.00213 | 0.886 |
| key: PUFA, polyunsaturated fatty acid; SNP, single-nucleotide polymorphism; SE, standard error | | | | | | | |
| a. Summary statistics for Alzheimer’s dementia from “Nat Genet 2019;51(3):404-413. doi: 10.1038/s41588-018-0311-9”. | | | | | | | |

**Table S3.** Distribution of phospholipid plasma fatty acids: the Atherosclerosis Risk in Communities (ARIC) Study

| **PUFA** | **Mean** | **SD** |
| --- | --- | --- |
| Linoleic acid (LA, 18:2n6) ^a^ | 21.99 | 2.69 |
| Arachidonic acid (AA, 20:4n6) ^a^ | 11.45 | 1.96 |
| α-Linolenic acid (ALA, 18:3n3) ^b^ | 0.14 | 0.05 |
| Eicosapentaenoic acid (EPA, 20:5n3) ^b^ | 0.56 | 0.30 |
| Docosapentaenoic acid (DPA, 22:5n3) ^b^ | 0.90 | 0.17 |
| Docosahexaenoic acid (DHA, 22:6n3) ^b^ | 2.82 | 0.88 |
| Key: PUFA, polyunsaturated fatty acid; SD, Standard deviation | | |
| a. Data source: “Am Heart J. 2008;156(5):965-74. doi: 10.1016/j.ahj.2008.06.017”. | | |
| b. Data source: “PLoS Genet 2011;7(7):e1002193. doi: 10.1371/journal.pgen.1002193”. | | |

**Table S4.** Sensitivity analysis of Mendelian randomization: phospholipid levels of polyunsaturated fatty acids and Alzheimer's Disease (21,982 clinically diagnosed cases and 41,944 controls)

| **PUFA** | **MR method** | **OR** | **(95% CI)** | **p** |
| --- | --- | --- | --- | --- |
| Linoleic acid (LA, 18:2n6) | IVW | 0.98 | (0.93, 1.03) | 0.658 |
|  | Weighted median | 0.98 | (0.93, 1.03) | 0.591 |
|  | MR-Egger | 0.96 | (0.88, 1.05) | 0.172 |
|  | MR-Egger (intercept) |  |  | 0.555 |
|  |  |  |  |  |
| Docosapentaenoic acid (DPA, 22:5n3) | IVW | 1.03 | (0.97, 1.09) | 0.375 |
|  | Weighted median | 1.02 | (0.96, 1.08) | 0.491 |
|  | MR-Egger | 0.99 | (0.88, 1.11) | 0.855 |
|  | MR-Egger (intercept) |  |  | 0.490 |
| Abbreviations: IVW=inverse variance weighted method; MR=Mendelian randomization; OR=odds ratio of 1 SD increase in plasma PUFA levels; PUFA=polyunsaturated fatty acid; 95% CI=95% confidence interval | | | | |

**Figure S1.** Mendelian randomization with MR-Egger method: phospholipid levels of linoleic acid and Alzheimer's Disease (21,982 clinically diagnosed cases and 41,944 controls)

**Figure S2.** Mendelian randomization with MR-Egger method: phospholipid levels of docosapentaenoic acid and Alzheimer's Disease (21,982 clinically diagnosed cases and 41,944 controls)

**Table S5.** Secondary analysis of Mendelian randomization: polyunsaturated fatty acids and Alzheimer's Disease using summary statistics of AD (including AD-by-proxy based parental diagnoses: 71,880 cases and 383,378 controls^a^)

| **PUFA** | **OR** | **(95% CI)** | **p** |
| --- | --- | --- | --- |
| Linoleic acid (LA, 18:2n6) | 0.997 | (0.992, 1.003) | 0.696 |
| Arachidonic acid (AA, 20:4n6) | 1.002 | (0.998, 1.006) | 0.461 |
| α-Linolenic acid (ALA, 18:3n3) | 0.995 | (0.982, 1.009) | 0.470 |
| Eicosapentaenoic acid (EPA, 20:5n3) | 1.002 | (0.987, 1.017) | 0.823 |
| Docosapentaenoic acid (DPA, 22:5n3) | 1.003 | (0.995, 1.011) | 0.477 |
| Docosahexaenoic acid (DHA, 22:6n3) | 0.997 | (0.965, 1.031) | 0.884 |
| OR=odds ratio of 1 SD increase in plasma PUFA levels; PUFA=polyunsaturated fatty acid; 95% CI=95% confidence interval | | | |
| a. Summary statistics for Alzheimer’s dementia from “Nat Genet 2019;51(3):404-413. doi:10.1038/s41588-018-0311-9”. | | | |
